# Comparing Deep Reinforcement Learning Algorithms’ Ability to Safely Navigate Challenging Waters

**DOI:** 10.3389/frobt.2021.738113

**Published:** 2021-09-13

**Authors:** Thomas Nakken Larsen, Halvor Ødegård Teigen, Torkel Laache, Damiano Varagnolo, Adil Rasheed

**Affiliations:** ^1^ Department of Engineering Cybernetics, Norwegian University of Science and Technology, Trondheim, Norway; ^2^ Mathematics and Cybernetics, SINTEF Digital, Trondheim, Norway

**Keywords:** deep reinforcement learning, autonomous surface vehicle, collision avoidance, path following, machine learning controller

## Abstract

Reinforcement Learning (RL) controllers have proved to effectively tackle the dual objectives of path following and collision avoidance. However, finding which RL algorithm setup optimally trades off these two tasks is not necessarily easy. This work proposes a methodology to explore this that leverages analyzing the performance and task-specific behavioral characteristics for a range of RL algorithms applied to path-following and collision-avoidance for underactuated surface vehicles in environments of increasing complexity. Compared to the introduced RL algorithms, the results show that the Proximal Policy Optimization (PPO) algorithm exhibits superior robustness to changes in the environment complexity, the reward function, and when generalized to environments with a considerable domain gap from the training environment. Whereas the proposed reward function significantly improves the competing algorithms’ ability to solve the training environment, an unexpected consequence of the dimensionality reduction in the sensor suite, combined with the domain gap, is identified as the source of their impaired generalization performance.

## 1 Introduction

In recent years, the development and interest in autonomous shipping have increased significantly. According to ICS (2020), shipping comprises around 90% of the world’s trade of goods. However, the mode of transport is not entirely free from accidents. Miscommunication and failure in judgments during navigation significantly contribute to the total number of accidents at sea caused by human error (Sánchez-Beaskoetxea et al., 2021). While their analysis of maritime accident reports between 1975 and 2017 indicates that accidents attributed to human error have a declining trend, their findings still show that the crew is responsible for 45.83% of all reported accidents at sea. Therefore, shifting the liability of safe operation from the human to an autonomous controller has excellent potential for improvements. With autonomy comes other challenges, but there is still a significant possibility to improve overall safety for autonomous ships compared to human-operated ships (Hoem et al., 2019). Autonomous ships can improve working conditions, lower damage-related and crew costs, and improve the ship’s environmental performance (NFA, 2020). However, this requires vessels capable of acting independently and handling unexpected changes in the environment on the fly.

The requirement for robustness and ability to handle challenging and potentially infinitely many situations make developing an autonomous vessel extremely challenging. Autopilot design for path following is a well-known discipline (Fossen and Blanke, 2000; Kim et al., 2015; Xiang et al., 2018) and has robust solutions using traditional methods (Bibuli et al., 2009). Significant challenges appear when we are to combine this with situational awareness and obstacle avoidance. With such an ample space of possible actions and strategies, explicitly programming the behavior is near infeasible and far from practical. Lattice-based path planners can robustly calculate kinematically feasible and collision-free trajectories in a discretized state space, as shown for ground vehicles in Cirillo (2017) and Ljungqvist et al. (2019). Such planners can balance the trade-off between path following and collision avoidance through reactive re-planning when new obstacles occur on the trajectory. However, all traditional approaches require are model-based, where the model derivation can be both time and resource-intensive. Fortunately, Reinforcement Learning (RL) (Sutton and Barto, 2018) is coming up as a promising alternative for autonomous control without the need for a dynamics model. The RL agent learns the end-to-end connection between observations and actions through the principle of trial and error, which has shown remarkable results in applications such as games (Silver et al., 2016), robotics (Niroui et al., 2019), autonomous vehicles (Kiran et al., 2021), process control (Nian et al., 2020), and industrial automation (Weigold et al., 2021). The main advantages of RL controllers are that they can be model-free, operate with arbitrarily abstracted control inputs, tackle high-dimensional state-spaces, and obviate the need for any explicit programming of comprehensive rules yet still learn the control law to fulfill the given objectives.

In the context of the current work, (Meyer et al., 2020b), (Meyer, 2020a) have shown the feasibility of applying Proximal Policy Optimization (PPO) RL algorithms to the dual-objective problem of navigating an underactuated surface vessel along a known path while avoiding collision with landmasses and dynamic obstacles along the way. The observation space of the RL agent is based on vessel guidance control theory and includes a novel feasibility pooling technique for reducing the dimensionality of a simulated high-density rangefinder suite used for obstacle detection. The marine vessel itself is based upon CyberShip II, as introduced in Section 2.1. After applying the PPO algorithm in a stochastic, synthetic environment (Meyer, 2020a), found that the trained agent perfectly generalized to multiple real-world scenarios simulating trafficked areas in the Trondheim fjord, Norway. Meyer et al. (2020a) expands on Meyer et al. (2020b) by hand-crafting a reward function that encourages the RL agent to comply with the International Regulations for Preventing Collisions at Sea (COLREGs) using the PPO algorithm. Havenstrøm et al. (2021) applies a curriculum learning technique with the PPO algorithm to control a 6-DOF underactuated autonomous underwater vehicle (AUV), gradually increasing the presence and severity of obstacles and disturbances during the RL training process. Grando et al. (2021) develops and compares two approaches based on the Deep Deterministic Policy Gradient (DDPG) and Soft Actor-Critic (SAC) RL algorithms, respectively, to navigate a simulated quadrotor drone to a target position in 3D, including air-water medium transitions. Overall, it is thus safe to say that the existing works in the available scientific literature prove the potential of RL in path following and collision avoidance with both stationary and moving obstacles. However, the presented works consider only a single (in one case two) RL algorithm to learn the control law. Historically, RL algorithms have been selected based on compatibility—e.g., discrete vs continuous state and action spaces and full vs partial observability of the environment—whereas modern RL algorithms are more generally applicable. Consequently, it is non-trivial to determine which RL algorithm is best suited for any given application.

Given the potential of model-free RL in autonomous control, this paper attempts to answer some significant issues that the existing literature has not yet answered to the best of our knowledge. More precisely, this study attempts to answer the following research questions:

• (Inter-comparison of different RL algorithms) how does PPO compare against competitive state-of-the-art RL algorithms?
• (Behavioral trade-offs between path following and collision avoidance) can task-specific metrics provide more insight into the learned policies?
• (Reward shaping in comparison to previous works) what is the significance of the reward function’s role in balancing between simultaneous multi-objective tasks?

The questions above are answered through multiple simulations and analyses, which are explained later on. Though the initial comparison may give the impression that one algorithm has a significant advantage, the subsequent analyses shed light on the importance of a particular responsibility when setting up the problem formulation.

This article addresses the research questions through five sections. Section 1, the current section, introduces the state-of-the-art relative to RL in autonomous control, motivates its potential in maritime applications, and presents the related work that forms the basis for this project. With this basis, the research questions hint toward the intended contribution of this work. Section 2 describes the relevant theory in the fields of vessel guidance and RL. Section 3 presents the simulation environments, reward functions and specifies the RL algorithms’ training configuration, evaluation, and comparison against each other for the dual-task of path following and collision avoidance. Section 4 shows the evaluation of the trained RL agents in both the training and real-world simulation environments. In addition to a general performance comparison, an analysis of the agents’ task-specific behaviors provides insight into how changing the reward function impacts the agents’ trade-off between path following and collision avoidance. Section 5 summarizes the findings in this project and hints toward future work.

## 2 Theory

This section gives an overview of the concepts required in the study. The purpose is not to give a complete detail of the concepts; hence the descriptions are short. However, references to the sources are provided when deemed necessary.

### 2.1 Vessel Guidance and Control

Ironically, though the RL algorithms applied in this work are considered model-free, a dynamics model of the vessel and its surrounding domain is necessary for training the RL agents in simulation compared to a direct real-world application. As the current state-of-the-art RL algorithms are generally too sample-inefficient and unpredictable during training, a direct application approach is infeasible. However, the well-researched theory in ship maneuvering has established that the symbolic representation of the dynamics of surface vessels is independent of the ship itself. It follows that the symbolic representation of the dynamics in full-scale is equivalent to that of a small-scale replica, and their distinctions lie in the numerical parameter values. The successful demonstration of an RL controller navigating the CyberShip II model in Meyer et al. (2020b) implies that the RL algorithms can navigate full-scale vessels.

CyberShip II is a 1:70 scale replica of a supply ship whose non-linear dynamics model was identified experimentally by Skjetne et al. (2004). As a fully actuated ship, it comes equipped with three actuators: propellers and rudders aft and a bow thruster fore. In theory, independent and simultaneous acceleration in each degree of freedom allows it to navigate any trajectory in its state space. However, at high speeds, the bow thruster becomes less efficient, and the ship becomes underactuated (Sørensen et al., 2017). Disabling the bow thruster input reduces the dimensionality of the action space while retaining sufficient and continuous control. The resulting control input, $f={\left[T_{u},T_{r}\right]}^{T}$, consists of the aft propellers’ thrust, *T*_*u*_, and the rudder moment in yaw, *T*_*r*_. With the control interface established, we introduce essential concepts in maritime guidance. These will later form the basis for the RL agent’s perception of its environment; the more informative navigation states we can engineer, the better the RL agent will learn a control law for the vessel.

#### 2.1.1 Coordinate Frames

Coordinate frames are necessary for describing the vessel’s position and dynamics relative to a reference on Earth. The North-East-Down (NED) reference frame, {*n*} = (*x*_*n*_, *y*_*n*_, *z*_*n*_), forms a tangent plane to the Earth’s surface. Intuitively, the positive direction of the *x*, *y*, and *z* axes point north, east, and down, respectively. The origin of the body frame, {*b*} = (*x*_*b*_, *y*_*b*_, *z*_*b*_), is fixed to the vessel’s position in {*n*}, and the *x*_*b*_ and *y*_*b*_ axes align with the ship’s longitudinal and transversal heading. Hence, the *z*-axis points down to complete the right-hand system.

#### 2.1.2 State Variables

Using the established reference and body frames, the generalized coordinates, $\eta ={\left[x^{n},y^{n},\psi \right]}^{T}$, describe the vessel’s position and yaw angle relative to {*n*}. The angle between the *x*_*n*_ and *x*_*b*_ axes defines the ship’s yaw angle, *ψ*. Correspondingly, ***ν*** = [*u*,*v*,*r*]^*T*^, describes the translational and angular velocities, where *u*, *v*, and *r* are the surge velocity, sway velocity, and yaw rate, respectively.

#### 2.1.3 Navigation

Path following is a natural control problem for marine vessels. Let **p**_*d*_(*ω*) = [*x*_*d*_(*ω*), *y*_*d*_(*ω*)] describe a parameterized path, where *x*_*d*_(*ω*) and *y*_*d*_(*ω*) are given in the NED frame. Then, $p_{d}\left(\bar{\omega }\right)$ describes the point on the path that minimizes the Euclidean distance to the vessel, where

$$\bar{\omega }=\arg\underset{\omega }{\min}{\left(x^{n}-x_{d}\left(\omega \right)\right)}^{2}+{\left(y^{n}-y_{d}\left(\omega \right)\right)}^{2},$$

which locally optimal solution can be calculated with the Newton-Raphson method using the previous estimate of $\bar{\omega }$ as the initial guess. It follows that the *Cross-Track Error* (CTE) between the path and the current position,

$$\epsilon =\left|\left|{\left[x^{n},y^{n}\right]}^{T}-p_{d}\left(\bar{\omega }\right)\right|\right|,$$ (1)

is a useful measurement for evaluating how far the vessel deviates from its path.

The *look-ahead reference point*, $p_{d}\left(\bar{\omega }+\Delta_{LA}\right)$, defines the point at a fixed distance, Δ_*LA*_, further along the path. This point is expressed as $p_{d}={\left[x_{d}\left(\omega \right),y_{d}\left(\omega \right)\right]}^{T}$ where *x*_*d*_(*ω*) and *y*_*d*_(*ω*) are given in the NED frame. It follows that the change in heading needed to navigate towards the look-ahead point, or *heading error*, is defined by

$$\tilde{\psi }=\text{atan}2\left(\frac{y_{d}\left(\bar{\omega }+\Delta_{LA}\right)-y^{n}}{x_{d}\left(\bar{\omega }+\Delta_{LA}\right)-x^{n}}\right)-\psi .$$ (2)

The *path angle*, *γ*_*p*_, relative to the NED frame can be parameterized by *ω*, such that

$$\gamma_{p}\left(\bar{\omega }\right)=\text{atan}2\left(y_{p}^{\prime }\left(\bar{\omega }\right),x_{p}^{\prime }\left(\bar{\omega }\right)\right),$$

where *x*_*p*_′ and *y*_*p*_′ are the first-order path derivatives. Finally, the *look-ahead heading error*

$${\tilde{\psi }}_{LA}=\gamma_{p}\left(\bar{\omega }+\Delta_{LA}\right)-\psi$$ (3)

defines the difference between the path angle at the look-ahead point and the current heading.

### 2.2 Reinforcement Learning

RL is one of the three major classes of machine learning frameworks in which an agent learns a policy to optimally react to its environment through trial and error, given only a scalar reward signal as feedback. This framework is advantageous in control problems where hand-crafting a control law is intractable or if a dynamics model is unobtainable. The RL problem describes as an optimization problem where the optimal solution is the policy whose parameters maximize the expected reward from acting in the environment (Sutton and Barto, 2018). The optimal set of parameters, θ*, can be expressed as:

$$\theta^{*}=\arg\underset{\theta }{\max}E_{\tau ∼\pi_{\theta }\left(\tau \right)}\left[\underset{t}{\sum }r\left(s_{t},a_{t}\right)\right],$$ (4)

where *θ* is the policy parameters, *τ* is the trajectory described by the set of states and actions, {*s*_1_, *a*_1_, … , *s*_*T*_, *a*_*T*_}, *π*_*θ*_(*τ*) is the trajectory distribution given by *θ*, and *r*(*s*_*t*_, *a*_*t*_) is the reward signal returned from the environment.

In the state-of-the-art, RL algorithms adopt methods from deep learning by utilizing neural networks to parameterize the RL policy, a practice that is called Deep Reinforcement Learning (DRL). As a sufficiently sized neural network may approximate any continuous function (Nielsen, 2015), these models are compatible with high-dimensional non-linear state spaces and thus suitable for complex decision-making policies. This work compares a set of the most commonly used, state-of-the-art, model-free RL algorithms. As the field of RL is advancing rapidly, there exist newer bleeding-edge algorithms than those presented in this work; such algorithms still have pending software implementations in trusted open-source software libraries. This study focuses on the evaluation and comparison of established algorithms rather than implementing the bleeding-edge. Thus, we summarize the relevant algorithms and try to unify the notation as much as possible.

#### 2.2.1 Reward Functions

All model-free RL algorithms aim to maximize the expected reward, as reward maximization lies at the core of the RL objective. Although Eq. 4 considers the total sum of rewards in expectation, it is common to scale down distant rewards by a *discount factor*, *γ* ^*t*−*t*^′ ∈ (0, 1], *t* > *t*′ ≥ 0, to adjust how greedy the optimal policy should be. One can consider the reward function as an implicit representation of the optimal policy and the discount factor as an induced “fear of death.” To clarify the latter, attributing high discounts to distant future (*t* ≫ *t*′) rewards implies that there is a risk that the agent may never reach that future state and should, instead, focus on the less discounted near future rewards.

However, reward signals seldom manifest naturally in the real world. It is, in most cases, necessary to manually design a reward function to encourage the desired optimal policy. *Sparse* rewards, i.e., presenting the agent with a reward only at each episode’s termination, induce the least amount of bias in the optimal policy. However, such sparsity can result in slow or non-convergent training in environments with long time horizons or challenging exploration.

In contrast, *dense* rewards provide the agent a reward at each time step in the environment, significantly improving the learning rate. To implement a dense reward function, the designer must have sufficient domain knowledge to shape relevant information in the environment into rewards. Consequently, dense rewards inject bias into the agent because the designer imposes a pre-existing notion of how the trained agent should act, whereas sparse rewards let the agent discover the optimal strategy itself.

#### 2.2.2 Off-Policy RL Algorithms

Off-policy RL algorithms discern between the current policy and the behavioral policy. The current policy is considered an estimate of the globally optimal policy given the collected transitions, (*s*_*t*_, *a*_*t*_, *r*_*t*_, *s*_*t*+1_), while the behavioral policy dictates how the agent acts in its environment. Transitions are collected into a replay buffer by the behavioral policy interacting with the environment. The replay buffer then acts as a non-parametric model of the dynamics in the environment. Decoupling the behavioral policy from the update rule enables off-policy algorithms to decorrelate sampled transitions from different behavioral policies for updating the current estimate of the global optimum.

#### 2.2.3 Deep Deterministic Policy Gradient

The Deep Deterministic Policy Gradient (DDPG) algorithm (Lillicrap et al., 2019) combines Q-learning (Watkins and Dayan, 1992), Deterministic Policy Gradient (DPG) algorithms (Silver et al., 2014), and Deep Q-Networks (DQN) (Mnih et al., 2015) to create a model-free, off-policy, actor-critic RL algorithm that can apply deep neural networks’ universal approximation property to control problems with continuous action spaces.

DDPG maintains two sets of model parameters. The primary network consists of an actor, *π*_*θ*_(*s*_*t*_), that deterministically maps the state, *s*_*t*_, to an action, *a*_*t*_, and a critic, *Q*(*s*_*t*_, *a*_*t*_), that evaluates the state-action pair (*s*_*t*_, *a*_*t*_). The target network contains slowly interpolated versions of the actor and critic, denoted *π*_*θ*′_(*s*_*t*_) and *Q*′(*s*_*t*_, *a*_*t*_), respectively. Specifically, the update rule is *θ*′ ← *τθ* + (1 − *τ*)*θ*′, where 0 < *τ* ≪ 1. These targets dampen the instability caused by the moving target problem, inherited from the original Bellmann equation, which updates the *Q*-function using bootstrapped targets from *Q* itself. A fixed-size, last in first out, replay buffer is used to store and decorrelate the samples and enable the algorithm to utilize its off-policy properties. Minibatches of transitions, (*s*_*i*_, *a*_*i*_, *r*_*i*_, *s*_*i*+1_), are uniformly sampled from the replay buffer when updating the networks’ parameters.

DDPG defines its behavioral policy as

$$\pi_{\theta }^{\prime }\left(s_{t}\right)=\pi_{\theta }\left(s_{t}\right)+N_{OU},$$

where the noise, $N_{OU}$, is sampled from an Ornstein-Uhlenbeck process. Alternatively, (Plappert et al., 2018) empirically shows that adding noise directly to the policy parameters results in more consistent exploration and a richer set of behaviors.

#### 2.2.4 Twin Delayed DDPG

The Twin Delayed DDPG (TD3) algorithm introduces a set of modifications to the DDPG algorithm to improve its baseline performance. Fujimoto et al. (2018) identifies that actor-critic RL algorithms that are based upon DPG inherit specific weaknesses in value-based RL methods. First, they show that the *overestimation bias* in Q-learning is present in DDPG and suggest a clipped variant of Double Q-learning to negate it. Independent **twin** Q-functions, $Q_{\theta_{1}},Q_{\theta_{2}}$, are trained in parallel and evaluated as an aggregated ensemble where the output is defined as the minimum value generated between the corresponding target Q-functions, $Q_{\theta_{1}^{\prime }},Q_{\theta_{2}^{\prime }}$, i.e.,

$$y=r+\gamma \underset{i=1,2}{\min}Q_{\theta_{i}^{\prime }}\left(s^{\prime },\pi_{\phi }\left(s^{\prime }\right)\right).$$

Both twins then use the same output, y, in their update rule.

Second Fujimoto et al. (2018), shows that the moving target problem leads to accumulating residual temporal difference error in the value estimates of deep function approximators and consequently suggests that frequent updates of the policy and target networks lead to divergent behavior. Reducing the frequency for updating the policy and target networks allows the critic to minimize its error before the targets are updated, which reduces the amount of variance induced by this error propagating through to the policy. In practice, the modification only requires **delaying** the policy and target network updates, such that they are updated once for every fixed number, *d*, of updates to the critic.

Finally Fujimoto et al. (2018), suggests *target policy smoothing* as a regularization strategy for deep value learning to reduce variance induced by overfitting inaccuracies in the value estimate of deterministic policies. Instead of calculating the target value for a single action, target policy smoothing bootstraps value-estimates with a small amount of noise added to the action:

$$\begin{array}{ll} y & =r+\gamma Q_{\theta^{\prime }}\left(s^{\prime },\pi_{\phi^{\prime }}\left(s^{\prime }\right)+\epsilon \right),\\ \epsilon & ∼\text{clip}\left(N\left(0,\sigma \right),-c,c\right), \end{array}$$

The noise, *ϵ*, is clipped within the constant interval, [−*c*, *c*], to keep the perturbed action close to the original action. This modification intends to lead the algorithm to attribute a higher value to actions that are robust to noise, which consequently results in safer policies in stochastic environments with failure cases.

#### 2.2.5 Soft Actor-Critic

Soft Actor-Critic (SAC) (Haarnoja et al., 2017) is a maximum-entropy, (soft) actor-critic, off-policy, model-free RL algorithm derived from soft policy iteration. This strategy introduces an entropy maximization term to the original RL objective (i.e., Eq. 4). Balancing the maximization of the reward and the entropy encourages the resulting policy to succeed at its task while acting as randomly as possible. This aids exploration even in the late phases of training, where other RL algorithms often converge to a sub-optimal solution due to the lack of exploration. Introducing the entropy term to the RL objective results in the following definition of the optimal policy parameters:

$$\theta^{*}=\arg\underset{\theta }{\max}E_{\tau ∼\pi_{\theta }\left(\tau \right)}\left[\underset{t}{\sum }r\left(s_{t},a_{t}\right)+\alpha H\left(\pi_{\theta }\left(\cdot |s_{t}\right)\right)\right],$$

where the temperature scaling term, *α*, determines the relative importance of the entropy term, $H\left(\pi_{\theta }\left(\cdot |s_{t}\right)\right)$, against the reward signal. Balancing this trade-off makes SAC particularly sensitive to reward scaling. However, Haarnoja et al. (2019) solves this by automatically regulating the temperature scaling term within an additional constrained entropy maximization problem that ensures that the policy’s entropy is larger than a lower bound. Revised versions of the SAC algorithm incorporate the clipped twin Q-function modification introduced in TD3 to mitigate the overestimation bias.

#### 2.2.6 On-Policy Algorithms

In contrast to off-policy RL algorithms, the current and behavioral policies are one and the same in on-policy RL algorithms. Therefore, the update rule expects data sampled from the current policy; updating the policy deprecates the existing sample of transitions, and new samples must be collected using the newest policy. Therefore, on-policy methods are generally less sample-efficient than off-policy methods, though they are more stable in return.

#### 2.2.7 Policy Gradient Methods

By deriving a cost estimator from Eq. 4, $J\left(\theta \right)=E_{\tau ∼\pi_{\theta }\left(\tau \right)}\left[r\left(\tau \right)\right]$, and differentiating it, $\nabla_{\theta }J\left(\theta \right)=E_{\tau ∼\pi_{\theta }\left(\tau \right)}\left[\nabla_{\theta }\log\pi_{\theta }\left(\tau \right)r\left(\tau \right)\right]$, we obtain the most basic policy gradient suitable for stochastic gradient descent. Modern algorithms have found more efficient policy gradients that have significantly reduced variance and sensitivity to reward scaling. Most state-of-the-art policy gradients utilize the Advantage estimator, *A* ^*π*^, which evaluates how good an action is compared to the average action in that state. Formally, the Advantage describes the difference between the expected reward-to-go, *Q* ^*π*^(*s*_*t*_, *a*_*t*_), and the value estimate, *V* ^*π*^(*s*_*t*_):

$$\begin{array}{ll} A^{\pi }\left(s_{t},a_{t}\right) & =Q^{\pi }\left(s_{t},a_{t}\right)-V^{\pi }\left(s_{t}\right),\\ Q^{\pi }\left(s_{t},a_{t}\right) & =\overset{T}{\underset{t^{\prime }=t}{\sum }}E_{\pi_{\theta }}\left[r\left(s_{t^{\prime }},a_{t^{\prime }}\right)|s_{t},a_{t}\right],\\ V^{\pi }\left(s_{t}\right) & =E_{a_{t}∼\pi_{\theta }\left(a_{t}|s_{t}\right)}\left[Q\left(s_{t},a_{t}\right)\right]. \end{array}$$

Instead of learning individual representations for *Q* and *V*, it is common to use the approximation *Q* ^*π*^(*s*_*t*_, *a*_*t*_) ≈ *r*(*s*_*t*_, *a*_*t*_) + *V* ^*π*^(*s*_*t*+1_), such that the discounted Advantage can be estimated using only the value function:

$$A^{\pi }\left(s_{t},a_{t}\right)\approx r\left(s_{t},a_{t}\right)+\gamma V^{\pi }\left(s_{t+1}\right)-V^{\pi }\left(s_{t}\right),$$

where *γ* is the discount factor.

#### 2.2.8 Proximal Policy Optimization

The Proximal Policy Optimization (PPO) algorithm (Schulman et al., 2017) simplifies the computationally expensive constraint calculations and second-order approximations in the Trust Region Policy Optimization (TRPO) algorithm (Schulman et al., 2015). The result is a model-free, on-policy, actor-critic RL algorithm similar to TRPO in stability and performance, easier to implement in code, and faster to execute. Common for both is that they optimize a *policy improvement objective*. Formally, the objective is to maximize the expected value of the discounted Advantage of the old policy, *π*_*θ*_, under the trajectory distribution of the new policy, *π*_*θ*′_:

$$J\left(\theta^{\prime }\right)-J\left(\theta \right)=E_{\tau ∼\pi_{\theta^{\prime }}\left(\tau \right)}\left[\underset{t}{\sum }\gamma^{t}A^{\pi_{\theta }}\left(s_{t},a_{t}\right)\right],$$

which is guaranteed to improve the policy between updates, but it is intractable in this form. In TRPO, (Schulman et al., 2015) proposes a surrogate objective using importance sampling to relax the inner expectation over actions and shows that the outer expectation over the state marginal can be relaxed as long as the updated policy remains “close” to the current policy. This closeness is estimated using the second-order Taylor expansion of the KL-divergence between the policies’ state marginal distributions and used to constrain the optimization problem in the TRPO algorithm by regulating the learning rate.

Schulman et al. (2017) proposes a relaxation of TRPO’s constrained optimization. By simply clipping the importance sampling term in the surrogate Advantage function to be close to unity, PPO prevents the policy update from diverging far from the previous policy. Alternatively, another variant of PPO applies dual gradient descent, adjusting the Lagrangian multiplier for the KL-divergence in reaction to a breach of the constraint. Although these modifications both allow the algorithm to break the constraint momentarily, PPO performs better in general than TRPO while having a significantly lower computational complexity.

## 3 Methodology

This section outlines the application of the RL algorithms presented in Section 2.2 to the simulation environments developed by Meyer et al. (2020b), Meyer et al. (2020a). We keep the naming convention of their original environment, MovingObstaclesNoRules, for consistency. Meyer et al. (2020a) demonstrates that PPO can solve the training environment and generalize to the simulated real-world environments with a 100% success rate. PPO will therefore act as a benchmark to compare against the competing algorithms: DDPG, TD3, and SAC. The following section describes the simulation environment, software, and hardware setup and how the different algorithms are evaluated and compared.

### 3.1 Simulation Environments

All environments used in this paper model calm ocean surfaces containing obstacles and paths, generated at random. At the start of the path, a surface vessel is initialized with a random heading. The vessel dynamics use the CyberShip II model parameters, whose 3-DOF surface model and accompanying assumptions are outlined in Meyer et al. (2020b). In each episode, the agent’s primary objective is to navigate the vessel along the path from start to end, while its secondary objective is to avoid the landmasses and other marine vessels obstructing its path as it goes. Episodes terminate when.

• the vessel is less than 50 m from the goal,
• the vessel’s progress along the path exceeds 99%,
• the vessel has collided,
• the agent has spent more than 10000 time-steps, or
• the cumulative reward becomes less than −2000.

A preliminary inspection led to the consideration that each competing RL algorithm performs poorly in the original MovingObstaclesNoRules training environment. To find why the algorithms struggle, we establish a set of training environments whose complexities range from trivial to original.

**FIGURE 1:**
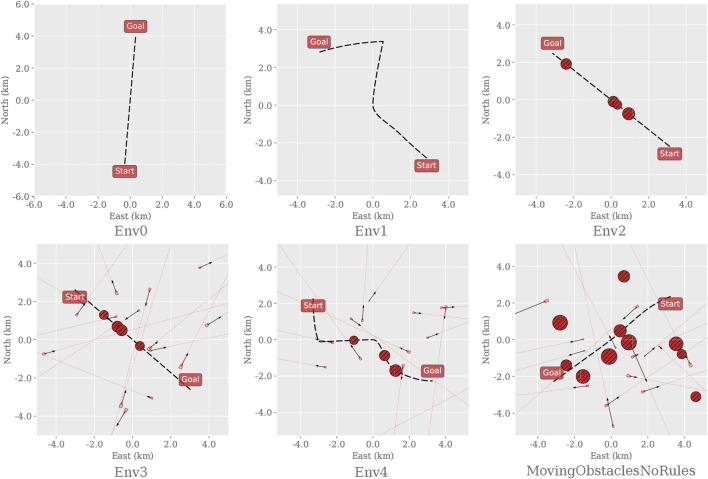
Instances of each training environment used to evaluate the applicability of different RL algorithms on the dual task of path following and collision avoidance. Before defining the path, the start and goal positions are initialized at random but with a fixed distance (8000*m*) apart. Env0, the trivial case, contains only a straight path. Env1 creates a curved path. Env2 inherits Env0 and places 4 static obstacles randomly along the path. Env3 inherits the properties of Env2 and adds 17 dynamic obstacles with random headings, sizes, and velocities around the path. Env4 inherits Env3 and adds curvature to the path. Finally, MovingObstaclesNoRules increases the number of static obstacles from 4 to 11 and scatters them randomly on and around the path; this represents the original training environment as defined in Meyer et al. (2020b).

We first consider two primary design elements that influence the environments’ complexity: the path curvature and the number, and placement, of static and dynamic obstacles that obstruct the path. Five additional simulation environments are created based on augmenting the complexity of the original. We let the first environment consist of a straight line with no obstacles to serve as the trivial case and increment the complexity, as described in Figure 1. The final and most complex training environment corresponds then to the original training environment defined in Meyer et al. (2020b). By distributing the task complexity across different training environments, starting at the trivial case, we aim to find the cut-off when there is a substantial difference in performance between the RL algorithms.

**FIGURE 2:**
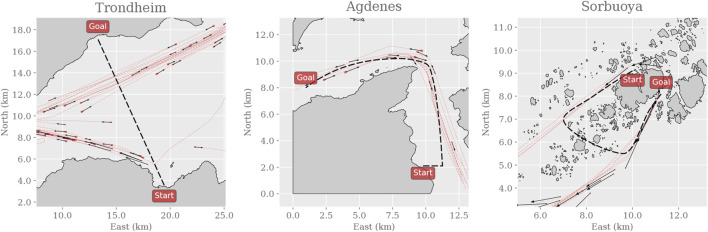
Snapshots of the three simulated real-world environments. Here, the paths and the landmasses are fixed for each instance of the environment, though each instance samples a random subset of the historical AIS data to create the dynamic obstacles. Thus, the dynamic obstacles are initialized with an initial position and velocity as shown with the black arrows and will follow the trajectories shown with the red dashed lines. As in the training environments, the agent’s goal is to safely navigate the vessel along the path from the starting position to the goal.

If the algorithms achieve similar performance in the most complex environment, three simulated real-world environments (Figure 2) will capture their ability to generalize to previously unseen and realistic scenarios. These environments are based on active routes in the Trondheim Fjord: we simulate the movement of the dynamic obstacles as dictated by historical AIS data and reconstruct the landmasses using terrain elevation data. The Trondheim environment consists thus of a straight path, unobstructed by landmasses but includes crossing traffic near the goal. The Agdenes environment consists of a curved path, unobstructed by landmasses but includes significant head-on and overtaking traffic along the path. The Sorbuoya environment consists of a curved path, heavily obstructed by landmasses, and includes head-on traffic in a narrow straight near the goal.

#### 3.1.1 Observation and Action Spaces

To learn a policy that optimizes the reward function, the RL agent must observe a partial representation of the environment that is rich enough to capture its most essential dynamics. The observation vector emitted from the environment is the concatenation of navigation and perception states. In the context of this paper, *navigation* refers to states that describe the vessel’s position, velocity, and orientation relative to the path, and *perception* refers to the rangefinder’s measured obstacle distances and obstacle velocities relative to the body frame. Table 1 describes the specific construction of the observation vector, where the definitions correspond to the vessel guidance theory in Section 2.1, and the FeasibilityPooling function is defined in (Meyer et al., 2020b). To influence its environment, the RL agent interfaces the two continuous actuators on the vessel as described in Section 2.1: the propeller thrust, *T*_*u*_, and the rudder moment in yaw, *T*_*r*_.

**TABLE 1:** RL agent’s observation vector at each time step *t* (Meyer et al., 2020a). The FeasibilityPooling algorithm calculates the maximum reachable distance in a given sector for the rangefinder suite. Obstacle velocities are relative to the body frame rotated such that the longitudinal axis is parallel to the centerline of the given sector and describe the velocity of the closest obstacle in that sector. Static obstacles assume a relative velocity of zero.

	Feature	Definition
Navigation	Surge velocity	*u* ^(*t*)^
	Sway velocity	*v* ^(*t*)^
	Yaw rate	*r* ^(*t*)^
	Cross-track error	*ϵ*^(*t*)^ (Eq. 1)
	Heading error	${\tilde{\psi }}^{\left(t\right)}$ (Eq. 2)
	Look-ahead heading error	${\tilde{\psi }}_{LA}^{\left(t\right)}$ (Eq. 3)
Perception	Obstacle closeness, first sector	$1-\frac{1}{S_{r}}\text{FeasibilityPooling}\left(x=\left\{x_{1},\ldots ,x_{d}\right\}\right)$
	⋮	
	Obstacle closeness, last sector	$1-\frac{1}{S_{r}}\text{FeasibilityPooling}\left(x=\left\{x_{N-d},\ldots ,x_{N}\right\}\right)$
	Obstacle velocity, first sector	[*v*_*x*,1_, *v*_*y*,1_]
	⋮	
	Obstacle velocity, last sector	[*v*_*x*,*N*_, *v*_*y*,*N*_]

#### 3.1.2 Reward Function

Despite the episodic and goal-conditioned nature of the simulation environment, sparse rewards are assumed infeasible due to the long time-horizon and the high number of possible failure cases per episode. Initially, we apply the dense reward function as implemented in Meyer (2020b). Equation. (5) specifies the reward function’s construction, and Table 2 describes its parameters.

$$\begin{array}{ll} r^{\left(t\right)} & =\left\{\begin{array}{cc} r_{\text{total}},\; & \text{if }r_{\text{total}}\geq 0\\ 2r_{\text{total}},\; & \text{if }r_{\text{total}}<0 \end{array}\right. \end{array}$$ (5)

$$\begin{array}{ll} r_{\text{total}}^{\left(t\right)} & =\left\{\begin{array}{cc} r_{\text{collision}},\; & \text{if collision}\\ \lambda r_{\text{path}}^{\left(t\right)}+\left(1-\lambda \right)r_{\text{colav}}^{\left(t\right)}+r_{\text{exists}},\; & \text{otherwise} \end{array}\right.\\ r_{\text{collision}} & =-\left(1-\lambda \right)r_{\text{coll}}\\ r_{\text{colav}}^{\left(t\right)} & =-\frac{\sum_{i=1}^{N}\frac{1}{1+\gamma_{\theta }|\theta_{i}|}S_{r}\exp\left(\gamma_{v}\max\left(0,v_{y}^{i}\right)-\gamma_{x}x_{i}\right)}{\sum_{i=1}^{N}\frac{1}{1+\gamma_{\theta }|\theta_{i}|}}\\ r_{\text{path}}^{\left(t\right)} & =\left(\frac{u^{\left(t\right)}}{2U_{\text{max}}}\cos{\tilde{\psi }}^{\left(t\right)}+\gamma_{r}\right)\left(\exp\left(-\gamma_{\epsilon }|\epsilon^{\left(t\right)}|\right)+\gamma_{r}\right)-\gamma_{r}^{2}\\ r_{\text{exists}} & =-\lambda \left(2\alpha_{r}+1\right) \end{array}$$

Based on the findings from the initial comparison, the reward function (Eq. 5) is reshaped to reduce its complexity while retaining enough information to guide the RL agent towards the desired policy. Since the reward is calculated at each time step, it is desirable to reduce its computational complexity. First, we remove the negative multiplier that scales the reward only if it is less than zero. Second, the RL agent receives a penalty both when colliding and being in the vicinity of obstacles. However, the vessel can arguably have a good trajectory while being close to obstacles. The critic network should learn to associate states where the vessel is close to an obstacle as lower-valued states through the collision penalty alone. Adding the penalty for closeness may accelerate this association, but it may also lead to a policy that is overly risk-avert early in training. Thus, we remove the *r*_colav_ term entirely from the reward function. Additionally, we decrease the collision penalty to *r*_coll_ = −1000, as the previous value of *r*_coll_ = −10000 can overshadow the penalty for deviating from the primary task of path following. Finally, we simplify the *r*_path_ term to:

$$r_{\text{path}}^{\left(t\right)}=\underset{\text{Speed term}}{\underset{︸}{\frac{u^{\left(t\right)}}{U_{\text{max}}}}}\cdot \;\underset{\text{Heading term}}{\underset{︸}{\frac{1+\cos\left({\tilde{\psi }}^{\left(t\right)}\right)}{2}}}\cdot \;\underset{\text{CTE term}}{\underset{︸}{\frac{1}{|\epsilon^{\left(t\right)}|+1}}}$$ (6)

Equation. (6) is well-behaved, has no hyperparameters, and all terms are bounded between 0 and 1. When the vessel is perfectly adhering to the path, moving at full speed, and in the right direction, then $r_{\text{path}}^{\left(t\right)}=1$. Setting the existence penalty *r*_exists_ = 1 makes zero the best possible reward in an episode. The proposed reward function can thus be described as:

$$r^{\left(t\right)}=\left\{\begin{array}{cc} r_{\text{coll}},\; & \text{if collision}\\ r_{\text{path}}^{\left(t\right)}-r_{\text{exists}},\; & \text{otherwise} \end{array}\right.$$ (7)

**TABLE 2:** Description of parameters in the initial reward function. The vessel’s maximum speed is denoted in decameters per second (1 dam = 10 m).

Scaling parameter	Interpretation	Value
*γ*_*ϵ*_	Cross-track error scaling	5.0
*γ*_*θ*_	Sensor angle scaling	10.0
*γ*_*x*_	Obstacle distance scaling	0.1
*α*_*r*_	Zero-reward relative speed	0.05
*r*_coll_	Collision reward	-10000
*γ*_*r*_	Constant multiplier	1.0
*λ*	Objective trade-off coefficient	0.5
Sensor parameter		
*U*_max_	Vessel’s maximum speed	1.0 dam/s
*N*	Number of sensors	180
*θ*_*i*_	Angle of sensor *i*	$-\pi +\frac{2\pi }{N}i$
*d*	Number of sensor sectors	9
*S*_*r*_	Sensor range	1.5 km
Δ_*LA*_	Look-ahead distance	3 km

### 3.2 Performance Evaluation

A trivial way to evaluate the algorithms is to compare their average accumulated reward, as reward maximization is at the core of each algorithm, and they share the same reward function. However, this will not provide much insight into the different behaviors that the agents may elicit. Instead, to develop understanding, we capture task-specific metrics from each episode, such as progress, collisions, time consumption, and cross-track error.

To find reasonable evaluation criteria, we first break down the RL agent’s task and analyze what is considered a success. From a logical perspective, the vessel’s task is to reach a destination with a fixed displacement from the start by navigating along a randomly curved path, while static obstacles are distributed on and around the path after the path’s creation. Additionally, dynamic objects (i.e., other vessels), each with random size and velocity, move around in the environment.

What are then the most important factors to consider? For instance, a vessel that never collides yet never reaches its destination is useless, though a vessel that adheres to the path but collides a few times has room for improvement. Given this intuition, we now move to discuss which metrics can differentiate between the trained RL agents and how to interpret them, in a per point fashion:

• **Can the vessel consistently navigate from the starting point to the goal?**

This can be measured by calculating the agent’s progress along the path, averaged over multiple rollouts in each environment. Progress is calculated as a percentage value by dividing the arc length between the starting position and $p_{d}\left(\bar{\omega }\right)$ (Section 2.1.3) by the total length of the path. An ideal agent will achieve an average progress score of 99 − 100*%*, whereas a lower score indicates that the agent either collides, gets stuck, or fails to follow the path. These failure modes are not captured by the progress score alone, thus necessitating additional task-specific metrics.

• **Does the vessel collide? If so, how often?**

An ideal agent will never collide. The collision rate (number of collisions per navigation task) is a decisive measure for the agents’ collision avoidance performance. Finding the collision rate is a simple matter of counting the number of collisions and dividing by the number of episodes. In the performance evaluation, the collision avoidance metric is defined as 100 ∗ (1 − *C*), where *C* ∈ [0, 1] is the collision rate of the agent. Thus, the collision avoidance scores 100 and 0% correspond to the collision rates 0 and 100%, respectively.

• **How well can the vessel adhere to its path?**

Considering that obstacles may obstruct the given path, sacrificing path adherence for collision avoidance is crucial for the agent to reach the goal. However, excessively avoidant behavior may result in significant deviations from the path or even total ignorance of the path-following task. On the other end, excessive path adherence may prevent the agent from taking the necessary detour around an obstruction. Section 2.1.3 introduced CTE as the minimum distance between the path and the vessel. Path adherence is calculated by normalizing the agents’ average CTE in a data-driven fashion and scaling them to a percentage value. This mapping of the CTE enables plotting the performance metrics on a common axis, whereas the CTE alone is an unbounded measure. Therefore, we say that a path adherence of 100 and 0% correspond to the minimum and maximum CTE achieved by any agent, respectively.

• **How much time does the agent spend on an episode?**

The time consumption (simulation steps) per episode measures whether the agent can make quick decisions, navigate at high speeds, and take the shortest deviating path, in contrast to navigating far away before heading towards the goal. This metric can differentiate between agents with similar progress scores by identifying agents that get stuck or fail to reach the goal by consistently exceeding the maximum number of time steps per episode. Thus, the time efficiency score is a percentage value, where 100 and 0% correspond to agents spending zero time steps and 10,000 time steps on average, respectively. Note, however, that agents with high collision rates may yield an artificially low time consumption.

The next step towards understanding how the environment complexity affects the performance indexes above for the various algorithms is to test the trained RL agents in their training environment and simulated real-world environments. We calculate metrics as averages over 100 episodes for each environment and for each algorithm to get statistically significant results. While the average progress metric primarily evaluates whether the agents solve the environment, the other metrics help differentiate among algorithms that perform similarly in the average progress sense. For the sake of brevity, in the following, we say that if the average progress exceeds 95%, the agent is said to have “solved” the training environment.

### 3.3 Simulation Software and Hyperparameters

Using the setup described above, we train four RL agents using the PPO, DDPG, TD3, and SAC algorithms as implemented in the Stable-Baselines Python library (Hill et al., 2018). All algorithms are executed on an AMD Ryzen 939,00X 12-core CPU and trained for a total of 1.5 million time steps each. The length of each simulation step is 1.0 s. Since the RL algorithms implemented by Stable-Baselines have varying parallelization capabilities, we do not compare the algorithms’ wall-time consumption.

For the sake of making this work repeatable by other authors, we summarize the non-default hyperparameters applied to the algorithms in Table 3. The remaining hyperparameters are left as their default values as defined by Stable-Baselines’ documentation (v2.9.0). SAC is the only RL algorithm that is applied using only the default hyperparameters. The source code used for executing the simulations is publicly available (Larsen, 2021).

**TABLE 3:** Non-default hyperparameters for each RL algorithm.

Hyperparameter	Description	Value
	Proximal Policy Optimization (PPO)	
n_steps	Number of steps to run for each env per update	1024
nminibatches	Number of training minibatches per update	32
lam	Bias vs variance trade-off factor for GAE (*λ*)	0.98
gamma	Discount factor (*γ*)	0.999
learning_rate	Learning rate	2e-4
	Deep Deterministic Policy Gradient (DDPG)	
memory_limit	Size of replay buffer	1,000,000
normalize_obs	Whether agent observations are normalized	True
gamma	Discount factor	0.98
actor_lr	Learning rate for actor network	0.00156
critic_lr	Learning rate for critic network	0.00156
batch_size	Size of the batch for learning the policy	256
action_noise	Action noise type and magnitude	OrnsteinUhlenbeck
		(*μ* = [0, 0], *σ* = [0.5, 0.5])
	Twin Delayed DDPG (TD3)	
buffer_size	Size of replay buffer	1,000,000
train_freq	Update the model every n steps	1000
gradient_steps	Gradient updates after each step	1000
learning_starts	Steps before learning starts	10000
action_noise	Action noise type and magnitude	N(μ=0,σ=0.1)
	Soft Actor Critic (SAC)	
None	All hyperparameters are default	N/A

## 4 Results and Discussion

### 4.1 Comparing the RL Algorithms in Environments of Increasing Complexity

As the preliminary investigation revealed that the off-policy algorithms struggle to solve the original training environment, this initial experiment attempts to find the point of failure for these algorithms. One agent is trained for each algorithm and environment (Figure 1) and tested accordingly. For instance, the PPO agent trained in Env0 gets evaluated in Env0 only. Though the training statistics would give a similar indication, the agents’ exploration strategies are typically suppressed in testing, making them behave greedily to exploit their estimation of the optimal policy.

**FIGURE 3:**
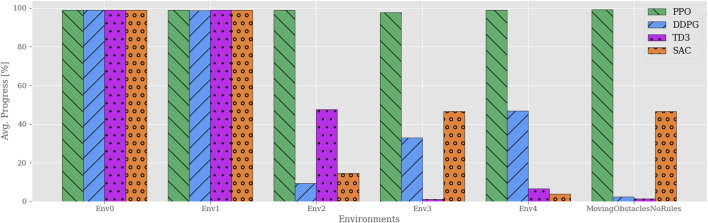
Average progress for all algorithms plotted vs environments of increasing complexity. Each agent is individually trained and tested in their respective environments, e.g., agents trained in Env0 are tested only in Env0. All the algorithms except PPO yield significantly reduced performance once static obstacles are introduced in Env2. Though neither off-policy algorithm exceeds 50% average progress, their results vary significantly among the environments.

To start this discussion, consider Figure 3, which compares the average progression of each algorithm in each environment. These results show that the PPO agents consistently solve each of their training environments regardless of the complexity. In contrast, the other algorithms are only capable of solving the trivial ones. In other words, once obstacles appear in Env2, none of the remaining algorithms manage to adopt policies with a reasonable trade-off between path following and obstacle avoidance. Though it is clear that the presence of obstacles is a common factor, the abrupt and volatile progress scores found for the off-policy RL algorithms imply that this metric alone does not capture a behavioral degradation as the complexity increases.

**FIGURE 4:**
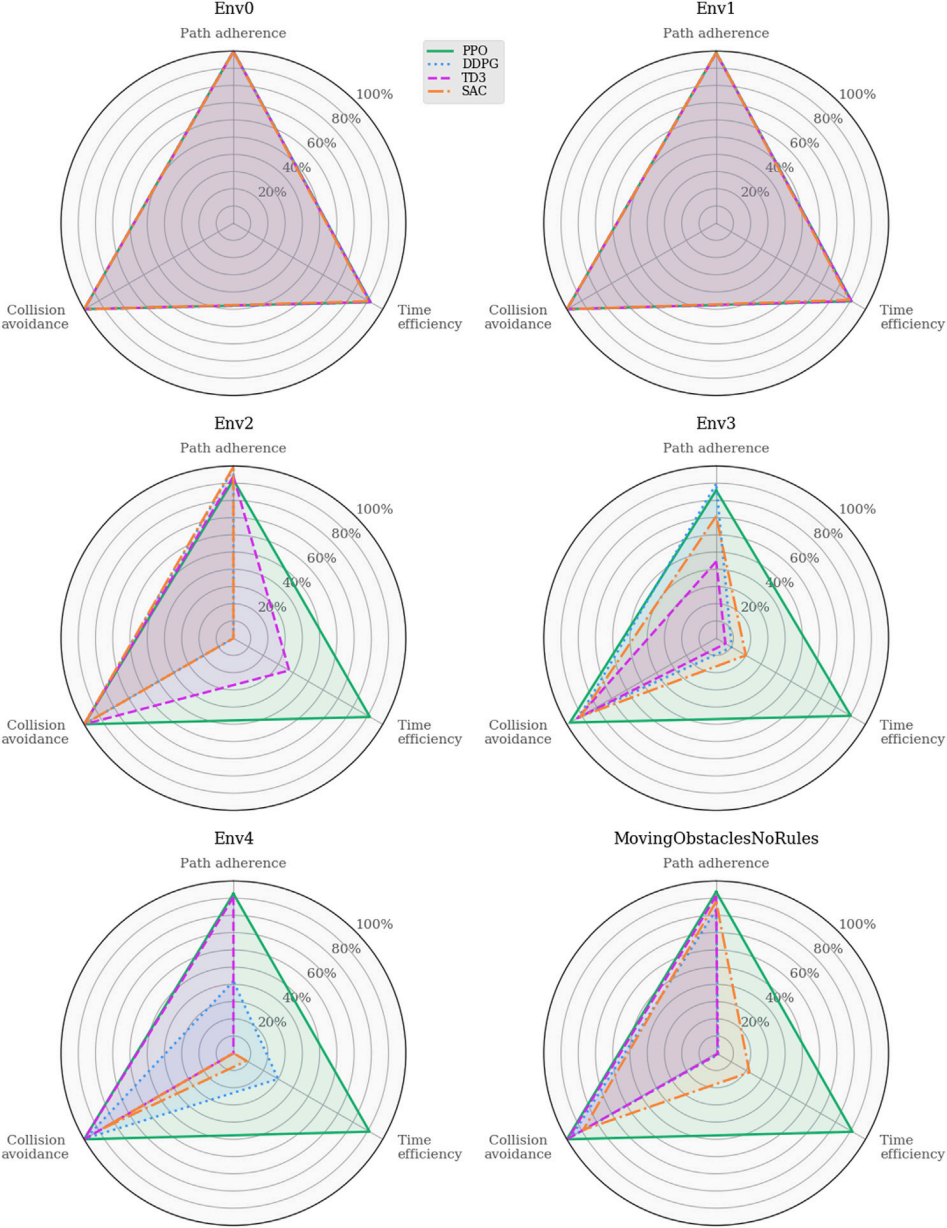
Performance analysis of task-specific metrics across the training environments. The indistinguishable behaviors in the trivial environments rule out critical software implementation faults for the added RL algorithms. Whereas the agents’ capacity for collision avoidance remains near-perfect across all scenarios, the time efficiency drops significantly for the off-policy algorithms in Env2 and subsequent training environments.

To further investigate the different behaviors of the learned policies, Figure 4 illustrates the task-specific metrics for each algorithm in each environment. As mentioned in Section 3.2, the path adherence metric maps the normalized CTEs to a percentage value in a data-driven fashion. DDPG produced the minimum CTE (3.7*m* in Env0), and SAC produced the maximum CTE (6091.6*m* in Env4). Thus, the path adherence scores shown in Figure 4 are calculated as $100-100*\frac{\text{CTE}-3.7}{6091.6-3.7}$. The agents’ behaviors are visually indistinguishable in Env0 and Env1, independently of the algorithm, likely because they find policies close to the optimal solution. Note that the PPO algorithm is producing agents that behave consistently across all environments. In Env2, both DDPG and SAC exceed the maximum number of time steps per episode on average, which, considering their near-perfect path adherence and collision avoidance, implies that the agents never deviate from the path and come to a standstill when encountering an obstacle. TD3 achieves similar performance in path adherence and collision avoidance but spends more than half of its time budget on average.

As intuition may suggest, the results also show that dynamic obstacles, introduced in Env3, clearly affect the agents’ path adherence scores; moreover, the simulations highlight a similar relationship between time efficiency and path progress as for Env2. In fact, there exists a consistent correlation between the path progress scores in Figure 3 and the time efficiency scores in Figure 4. In contrast, all algorithms yield near-perfect collision avoidance scores regardless of their agents’ ability to progress along the path. This particular consistency in behavior may suggest that the collision avoidance mechanisms included in the problem formulations have the most influence in shaping all the various final policies learned by the algorithms.

**FIGURE 5:**
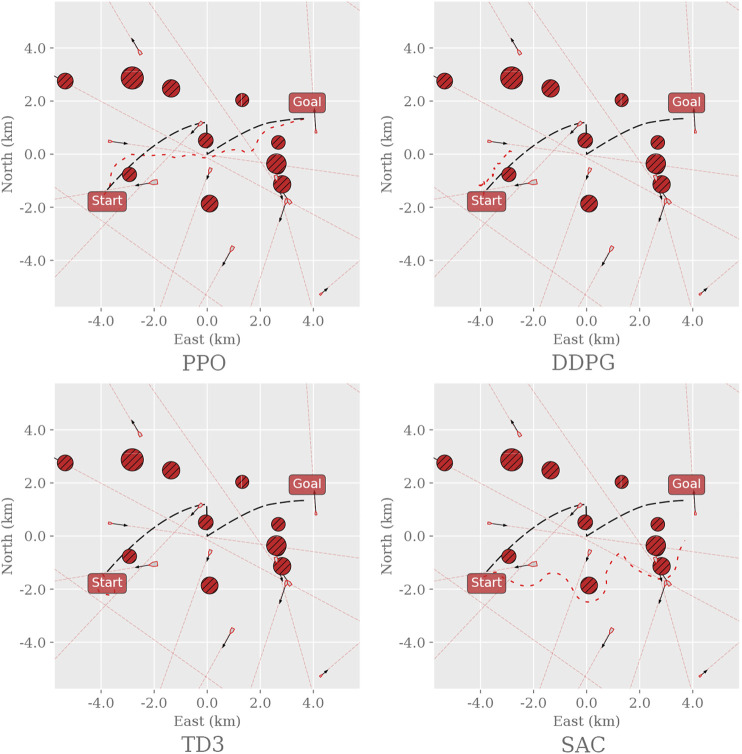
Sample trajectories for the different RL agents in the MovingObstaclesNoRules environment. Black dashed line: path to follow; red dashed line: path taken by the vessel. The environment was generated and sampled equally for all algorithms by setting the random seed to zero and sampling the first episode.

Figure 5 shows a randomly sampled trajectory for each RL algorithm tested in the MovingObstaclesNoRules environment. The PPO agent reaches the goal by loosely following the path; DDPG halts after passing the first obstacle; TD3 remains inert at the starting point; SAC explores wildly while moving in the general direction of the goal.

**FIGURE 6:**
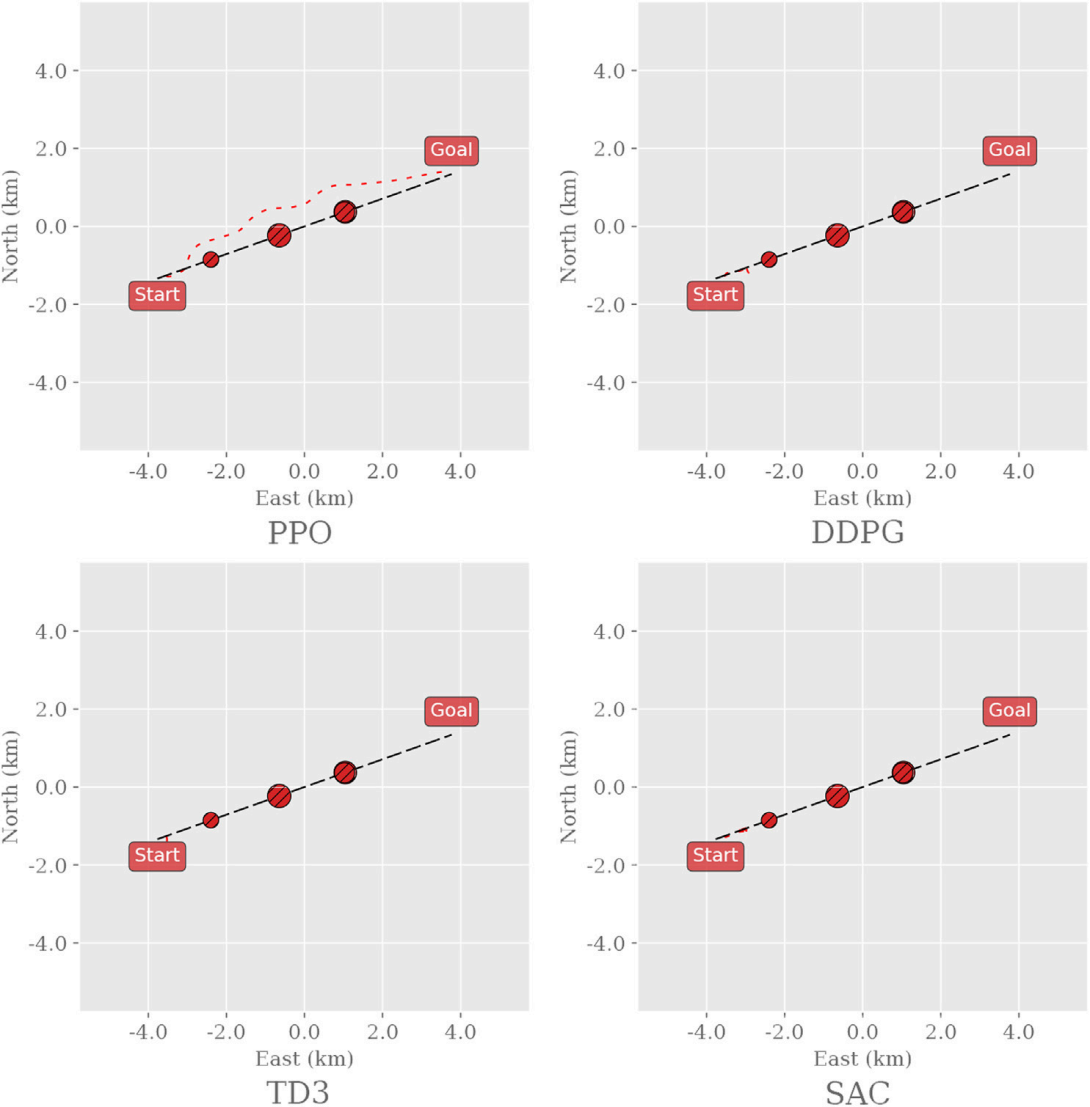
Sample trajectories for the different RL agents in the Env2 environment. Black dashed line: path to follow; red, dashed line: path taken by the vessel. The environment was generated and sampled equally for all algorithms by setting the random seed to zero and sampling the first episode.

Figure 6 shows a random sample of the agents’ paths taken in Env2. PPO is the only agent that circumvents the obstacles and reaches the goal, whereas Both DDPG and SAC stop moving when they encounter an obstacle, and TD3 remains inert at the starting point. Neither agent collides; the off-policy algorithms rather choose to remain still and wait for 10,000 time steps until the episode ends.

Although there is a possibility of inadequate hyperparameter tuning, the unwavering collision avoidance scores in Figure 4 might indicate that the reward function is over-engineered for this behavior in particular. Aggregating the penalties for collisions and obstacle closeness may discourage the agents from assuming any policy that allows the vessel to be in the vicinity of an obstacle. Agents might therefore ignore their primary task: following the path and reaching the goal. For off-policy RL algorithms, in particular, these considerations imply that the replay buffer hardly ever contains any transitions close to the finish, such that the critic never learns that the agent can collect better rewards by balancing the collision avoidance and path following tasks. Ultimately, this may be a testament to PPO’s strong exploration strategy and robust trust-region-based policy update strategy.

**FIGURE 7:**
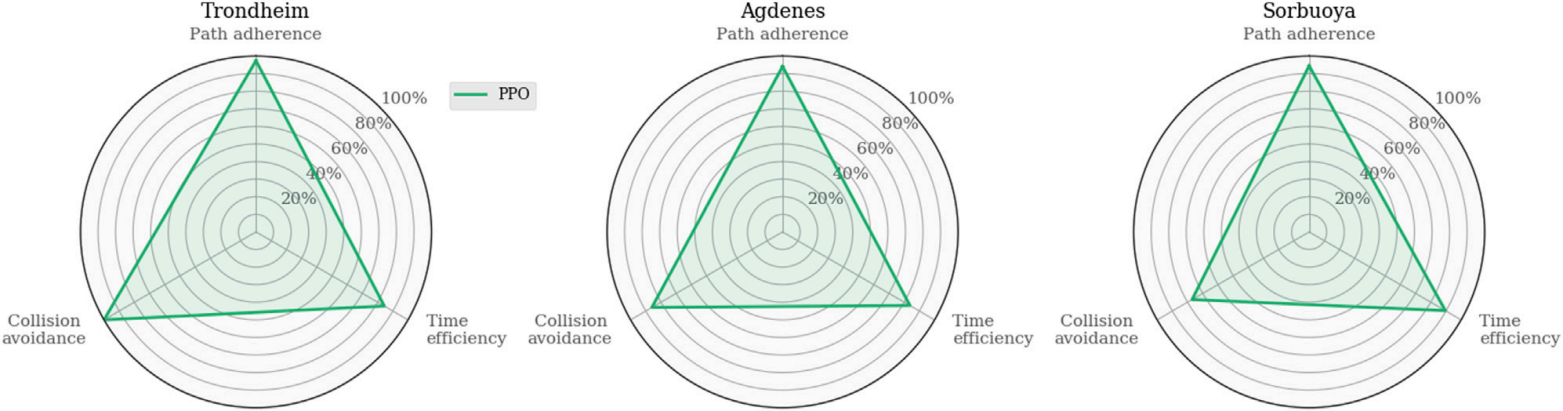
Evaluating the PPO agent trained in MovingObstaclesNoRules by testing it in simulated real-world environments for later reference.

To discuss PPO in more detail later on, consider that the corresponding agent trained in MovingObstaclesNoRules is tested in the simulated real-world environments (Figure 2) to act as a reference. As expected, Figure 7 illustrates that the PPO agent generalizes well and exhibits graceful degradation in collision avoidance.

### 4.2 Performance Comparison After Reward Shaping

**FIGURE 8:**
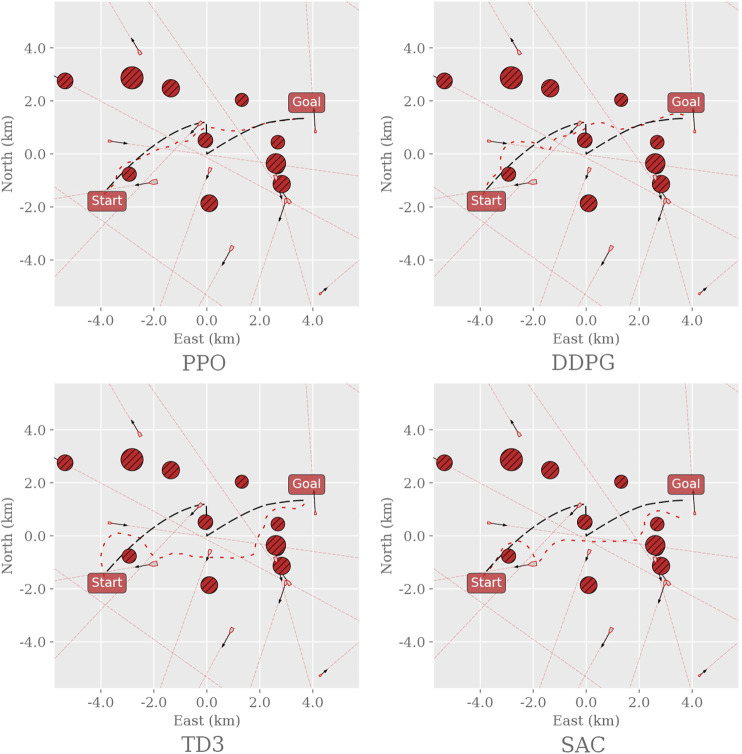
Sample trajectories from the different RL agents acting in the MovingObstaclesNoRules environment. The agents shown was trained using the simplified reward function. Black dashed line: path to follow; red dashed line: path taken by the vessel. The environment was generated and sampled equally for all algorithms by setting the random seed to zero and using the result from the initial episode.

**FIGURE 9:**
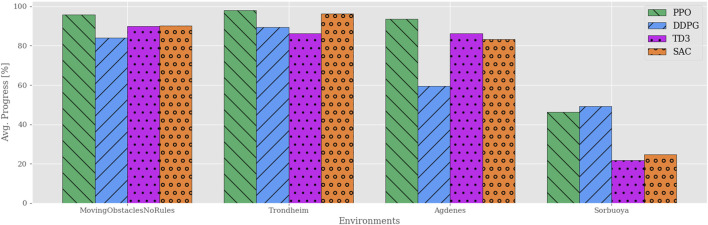
Path progression performance comparison between RL algorithms in training and real-world simulation environments using the simplified reward function. Compared to the previous reward function, the off-policy RL algorithms show a drastic increase in performance. In contrast, PPO now performs considerably worse in the Sorbuoya environment, yet maintains its performance in the other testing environments and is still the best performer overall.

After training the agents in the MovingObstaclesNoRules environment using the simplified reward function in Eq. 7, each agent is evaluated in both the training environment and the real-world simulation environments. Figure 8 shows sampled test trajectories from the training environment, and Figure 9 shows the average progress made for each agent. Compared to the previous results (Figure 3), the off-policy RL algorithms show a significant improvement in path progression. In particular, the DDPG and TD3 agents’ average progress increases from near-zero to 84*%* and 90*%*, respectively. These improvements are reflected in the sample trajectories, where all algorithms exhibit improved path adherence. Even PPO exhibits visibly improved path adherence in this scenario compared to its previous trajectory in Figure 5. Despite these improvements, neither of the off-policy RL algorithms meet the pre-specified minimum criteria of 95*%* average progress and are therefore not considered to have sufficiently solved the training environment. Testing the agents in the simulated real-world environments shows that most algorithms generalize well by maintaining their achieved level of performance in the training environment. However, their scores decline significantly in the Sorbuoya environment. DDPG is an exception considering its performance drop in the Agdenes environment while emerging as the best performer in Sorbuoya. Following the behavioral analysis, Section 4.3 provides an in-depth discussion of this impaired generalization performance.

PPO’s scores suffered slightly overall, though most notably in the Sorbuoya environment. Considering that all hyperparameters remain unchanged from the previous setup, it is not surprising to observe a slight reduction in PPO’s performance. RL algorithms are indeed notoriously sensitive to changes in hyperparameters, which must be tuned specifically to the relevant problem setting. Moreover, tuning the hyperparameters for the other RL algorithms may at least theoretically raise their performance to match, or even surpass, PPO. Despite this theoretical possibility, no attempt to tune the algorithms was made due to a lack of a systematic approach to finding optimal hyperparameters.

**FIGURE 10:**
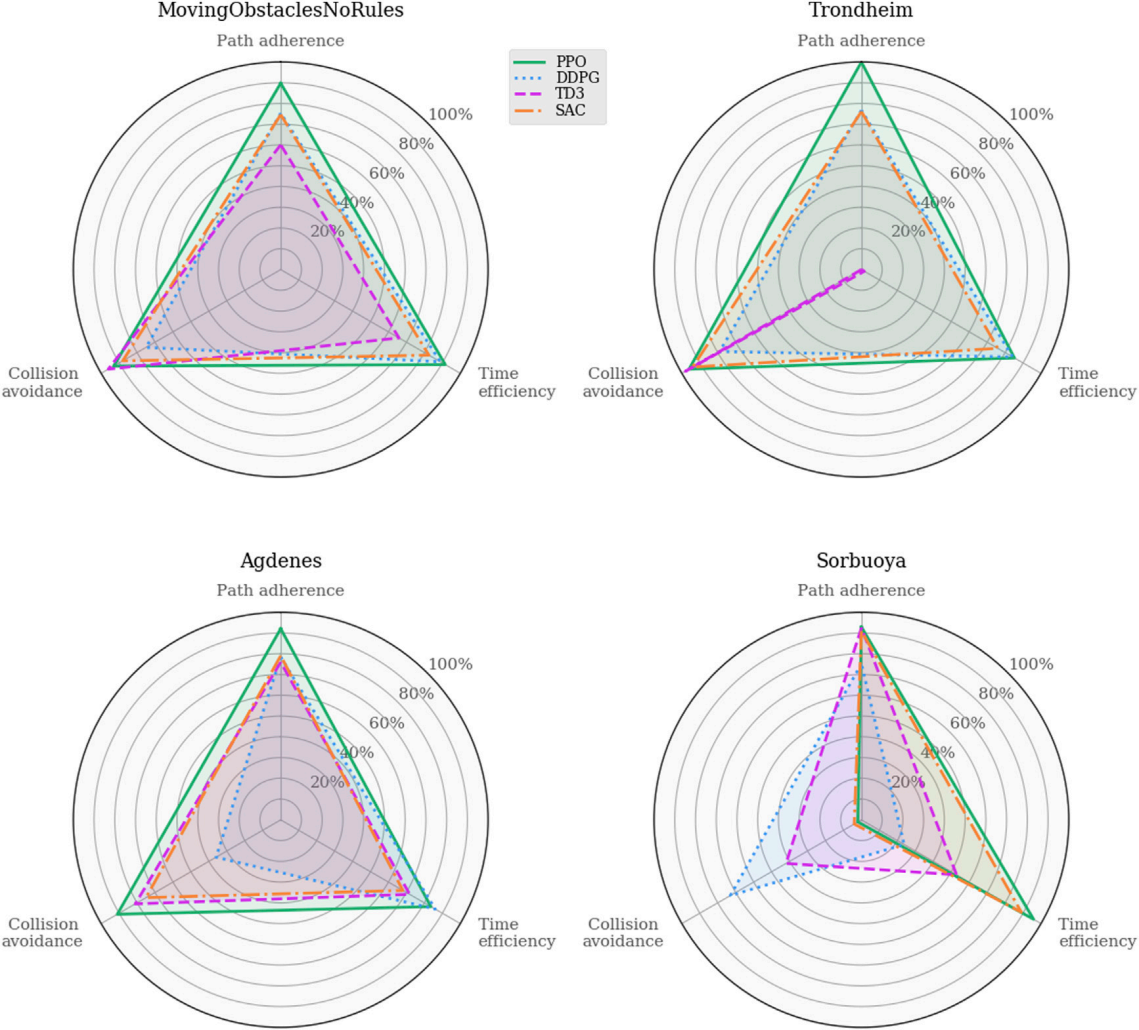
Performance analysis of task-specific metrics across simulation environments. These results provide insight into the agents’ trade-off between the path following and collision avoidance tasks. Their behaviors are similarly well-rounded in MovingObstaclesNoRules, and the discrepancies start to show when the agents are generalized to real-world environments.

Analyzing these agents’ path following and obstacle avoidance behaviors are of particular interest due to the intentionally omitted closeness penalty in this iteration of the reward function. Figure 10 shows the task-specific performances in the training and real-world environments. Again, the path adherence scores are mapped to a percentage value using the minimum CTE (52.2*m*, PPO in MovingObstaclesNoRules) and the maximum CTE (1868.5*m*, TD3 in Trondheim): $100-100*\frac{CTE-52.2}{1868.5-52.2}$.

**FIGURE 11:**
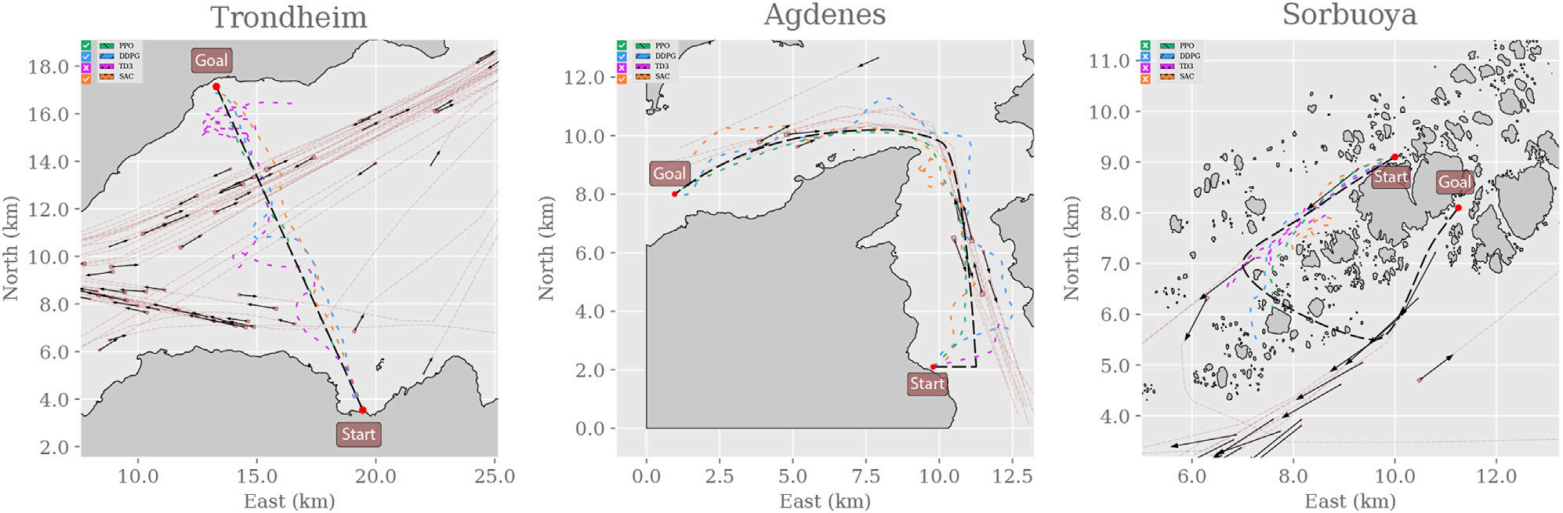
Trajectories from evaluating the RL agents in each real-world simulation environment were sampled from the first episode using a fixed random seed (zero). Therefore, the generated traffic is equivalent between each algorithm, allowing the trajectories to be overlaid and color-coded to minimize the number of subplots. Checkmarks highlight whether the agents reach the goal state in the environment. PPO, DDPG, and SAC succeeded in both Trondheim and Agdenes. TD3 failed in all scenarios, and all algorithms failed in Sorbuoya. In Trondheim, PPO is consistently following the path from start to end. SAC exhibits some drifting to the east, but its trajectory is significantly less erratic than DDPG and TD3. It seems that both DDPG and TD3 avoid a crossing vessel and return to the path after the interaction. DDPG eventually reaches the goal, whereas TD3 appears too collision-avert to approach the coast and reach the goal. In Agdenes, PPO maintains the trajectory closest to the path, DDPG crosses the traffic and ends up maneuvering a lot to avoid collisions, TD3 collides almost instantly with a tiny vessel, and SAC generally keeps a steady course but struggles to navigate around the opening of the fjord before succeeding. In Sorbuoya, PPO and DDPG progress slightly further than TD3 and SAC, who travel back and forth looking for an entry. However, neither agent manages to find a path through the densely packed islands.

Whereas Figure 9 shows that all algorithms achieve somewhat similar progress scores in MovingObstaclesNoRules, the behavioral analysis (Figure 10) reveals PPO’s superior path adherence capability without sacrificing collision avoidance or time efficiency. Since this environment contains both static and dynamic obstacles, perfect path adherence is impossible. In contrast, the Trondheim has few obstructions other than the crossing traffic, making perfect path adherence possible. While the algorithms still perform similarly in path progress, PPO significantly outranks the competing algorithms in path adherence. On the opposite end, the near-minimum time efficiency and path adherence of TD3 indicates that it never actually reaches the goal at all. Considering the accompanying near-perfect collision avoidance performance and that the end of the path in Trondheim is close to land, the TD3 agent likely prevents itself from reaching the goal due to a higher affinity towards collision avoidance. Figure 11 highlights this behavior.

In the Agdenes environment, DDPG struggles with obstacle avoidance, which is reflected in its progress score in Figure 9, though its path adherence is on par with TD3 and SAC. Its time efficiency score is artificially high due to the collision rate. PPO, TD3, and SAC show well-rounded behaviors, though PPO outperforms them on all accounts by approximately 10%. Sorbuoya, as the most challenging real-world scenario, brings out the most deviation in collision avoidance abilities. Interestingly, PPO is now the worst performer in this category and follows close after DDPG as the new best performer in average progress. Ultimately, none of the RL algorithms introduced to compete against PPO sufficiently solved the synthetic training environment using either reward function. The following section discusses possible reasons why the changes in the reward function facilitate better results in training for a broader range of RL algorithms, yet they produce agents with significantly reduced generalization performance compared to the previous one.

### 4.3 Implications of the Differences in Learned Policies

Compared to the previous reward function, the new iteration of the reward function is sparser by nature; thus, agents need to collide more before fully comprehending the risks involved when acting around obstacles. Therefore, the agents’ underdeveloped collision avoidance strategies could result from sparsifying the reward function without increasing training time. As for path following, it is clear that the off-policy agents are uninhibited by excessive risk-aversion and correctly prioritize their tasks, i.e., they now primarily focus on path following and learn to improve that skill via collision avoidance as a secondary priority. Ultimately, the task priorities are better balanced, though the speed of convergence appears to be reduced. However, these results warrant a thorough inspection of the relevant underlying mechanics in the environments.

Intuitively, one might think that Sorbuoya is the closest environment to the training environment. However, there is a particular reason why this is not the case. Recall that Trondheim serves as the trivial case, where the challenges consist only of a goal near land and some crossing traffic that may or may not interfere with the vessel. Agdenes spices things up slightly by introducing head-on traffic, inevitably navigating within the vessel’s sensor suite range. Otherwise, the path remains unobstructed by landmasses, and the goal is far from land. Sorbuoya, similar to the training environment, has an extensively obstructed path and modest traffic overall. Thus, intuitively, the agents should manage to navigate between the islands but perhaps run into trouble in the narrow straight with head-on traffic. However, there is a significant scaling-based domain gap between the training and testing environments. Whereas obstacles in the training environment are Poisson distributed with a mean radius of 300 m, Sorbuoya contains islands down to approximately 30–50 m in diameter. Similarly, the widths of the synthetic dynamic vessels are Poisson distributed with a mean of 100m, whereas the size of the real vessels are defined from their overall lengths found in the AIS data, ranging from 19 m (ship: Multi Innovator) to 299.7 m (ship: Golden Horizon), and the mean ship length is 59.6 m. Consequently, obstacles in Trondheim, Agdenes, and Sorbuoya are tiny in comparison to the training environment.

To understand the implications of this domain gap, we underline the signal processing applied to the vessel’s sensor suite. The RL agent relies on its observation space to detect and react to obstacles near the vessel. Recall that the perception vector (Table 1) contains, for each sector, a measure of obstacle closeness, i.e., the maximum reachable distance, as well as the relative velocity of the nearest dynamic obstacle (if any). The significantly smaller obstacles in the simulated real-world environments must be proportionally closer to the vessel before the FeasibilityPooling algorithm reduces the perceived maximum reachable distance in the corresponding sector, compared to the training environment. Therefore, the perception vector will produce sudden jumps in sector closeness; when a small obstacle is close enough to block a sector, then that sector’s maximum reachable distance jumps from *S*_*r*_ to the distance to the obstacle. The vessel may start an evasive maneuver, only to experience that the obstacle disappears from its perception when in the intersection of two sectors. Following the central assumption that the dynamics follow a Markov Decision Process (MDP) and the fact that the applied neural network architecture does not accommodate temporal aspects of the environment (not using recurrent neural networks or temporally stacked instances of observations), the agent will subsequently act as if the obstacle was never there. In other words, the vessel is oblivious of smaller obstacles until they are very close compared to the ones in the training environment. At medium range, they may pop in and out of existence. Though velocity observations are part of the observation vector, apparently, this only applies to the closest dynamic obstacle in each sector, and static obstacles assume a zero relative velocity. As a result, static obstacles can be imperceptible within the range of the sensor (*S*_*r*_). Whereas dynamic obstacles within sensor range may be invisible in terms of distance, the agent can perceive whether there is a vessel *somewhere* in that sector.

With these considerations in mind, the closeness penalty in Eq. 5 likely plays a larger role than just accelerating the agent’s understanding of the collision risks. The closeness penalty, in effect, drives the agent to adopt a policy that maximizes the distance to any obstacle while following the path. This behavioral aspect would provoke stronger reactions to obstacles suddenly appearing in the vicinity than the corresponding behavior encouraged by the proposed reward function that penalizes collisions only. Therefore, the closeness penalty makes the RL agent more robust against the blind spots in its perception, which is reflected in PPO’s higher generalization performance before the reward shaping (Section 4.1). In summary, it is likely that the combination of the domain gap, the perceptual blind spots, and the relaxed and sparser reward function ultimately leads to a more challenging generalization problem for the RL agent than previously anticipated.

## 5 Conclusion and Future Work

We provided an inter-comparison between common state-of-the-art RL algorithms applied to a continuous control problem in which the balancing between two independent tasks of path following and collision avoidance is critical for achieving good performance. Though the initial comparison gave the impression that the PPO algorithm had a significant advantage over DDPG, TD3, and SAC, the subsequent behavioral analyses shed light on the designer’s role in implicitly influencing the optimal policy through the construction of the reward function. An iteration of reward shaping attempted to balance the rewards and penalties given for the path following and collision avoidance tasks. Although the second generation of off-policy RL agents performed significantly better in the training environment and exhibited more well-rounded behavioral characteristics, removing the closeness penalties uncovered a potential issue with the established observation space given to the RL agents.

The main conclusions from the current study can be itemized as follows

• The primary purpose of this paper was to benchmark the applicability of competing state-of-the-art RL algorithms on the dual task of path following and collision avoidance. To do so, we challenged the performance of PPO in a continuous control problem, for which it was known to solve, with three off-policy RL algorithms. Comparing the average progress of each trained agent resulted in PPO significantly outperforming the competitors.
• Comparing the RL agents using their progress scores alone was insufficient to understand why the off-policy algorithms struggled to solve their training environment. The task-specific analyses helped to highlight the key aspects of the agents’ behavior that negatively impacted their performance. In particular, all agents exhibited near-perfect collision avoidance performance regardless of their ability to progress along the path in any environment. However, this exaggerated affinity toward collision avoidance prevented the off-policy RL algorithms from performing their primary path following task in the environment.
• The behavioral analysis inspired scrutinizing the role of the reward function in balancing the affinity between the path following and collision avoidance tasks. An iteration of reward shaping without penalties for obstacle closeness yielded a significant improvement in average progress and well-rounded task-specific metrics for the off-policy RL algorithms. Though their generalization performance exhibited an initially graceful degradation in performance, an unexpected consequence of the rangefinder’s dimensionality reduction algorithm, combined with the domain gap between the training and real-world environments, is identified as the most likely source for their impaired generalization performance. Although PPO suffered from a slightly degraded generalization performance, it emerged again as the best performing algorithm.

Although the current paper demonstrated the potential of RL-based approaches for addressing the complex dual objective of path following and collision avoidance, several shortcomings still need to be addressed. The first one stems from the fact that one of the advantages of RL-based approaches is that they can be model-free; however, in this work, we had to use a model because the training was conducted in a purely synthetic environment. This setup is necessary when implementing the current black-box approaches, as the RL agents can only learn collision aversion through directly experiencing the consequences of colliding. Training like this directly on a real-world system is both excessively costly and time-consuming, besides risky. To address this issue, one can utilize the concepts of predictive safety filters (Wabersich and Zeilinger, 2021), enabling the transfer of the techniques presented in this paper into real-world applications through the explicit consideration of state and input constraints. Another shortcoming of the work is that the effects of wind, waves, and currents were completely ignored. This, in combination with the surface model assumption, comprises a significant domain gap between the simulated and real settings. Consequently, the controllers obtained in this work are unlikely to transfer well to a physical CyberShip II model set to cross the Trondheim fjord. However, implementing these effects is not seen as a major challenge, as demonstrated in Havenstrøm et al. (2021) in the case of ocean current disturbances on RL-controlled autonomous underwater vehicles with six degrees of freedom. Lastly, the lack of interpretability and explainability of neural network policies need to be addressed before the proposed approach can be considered an alternative to traditional controllers. Symbolic regression, which has been used to discover hidden physics from data (Vaddireddy et al., 2020), can potentially convert the trained policies into human-interpretable control laws. Although these challenges currently prevent us from truly utilizing the model-free property of RL controllers, they can be addressed and solved in simulation and guide the RL framework towards being safe and robust when applied to physical applications.

The field of RL is advancing quickly, and novel algorithms have emerged after SAC (being the youngest algorithm in this set). However, the bleeding-edge RL algorithms have not yet been implemented in user-friendly open-source Python libraries and benchmarked against well-established algorithms. While it would be desirable to include bleeding-edge algorithms, this work focuses on evaluating RL algorithms with robust and user-friendly software implementations. Historically, modern RL algorithms are becoming increasingly applicable to a broader range of problem settings, and their performance is increasing proportionally. Thus, future RL algorithms will inevitably prove to outperform any of the ones considered in this work.

## Data Availability

The raw data supporting the conclusions of this article will be made available by the authors, without undue reservation.
